# Synergistic suppression of autoimmune arthritis through concurrent treatment with tolerogenic DC and MSC

**DOI:** 10.1038/srep43188

**Published:** 2017-02-23

**Authors:** Rong Li, Yujuan Zhang, Xiufen Zheng, Shanshan Peng, Keng Yuan, Xusheng Zhang, Weiping Min

**Affiliations:** 1 College of Basic Medical Sciences and Institute of Immunotherapy of Nanchang University, and Jiangxi Academy of Medical Sciences, Nanchang, China; 2 Jiangxi Provincial Key Laboratory of Immunotherapy, Nanchang, China; 3 Departments of Surgery, Pathology, and Oncology, University of Western Ontario, London, Canada

## Abstract

Rheumatoid arthritis (RA) is an autoimmune disease characterized by progressive immune-mediated joint deterioration. Current treatments are not antigen specific and are associated with various adverse. We have previously demonstrated that tolerogenic dendritic cells (Tol-DC) are potent antigen-specific immune regulators, which hold great promise in immunotherapy of autoimmune diseases. In this study, we aimed to develop new immunotherapy by combining Tol-DC and mesenchymal stem cells (MSC). We demonstrated that RelB gene silencing resulted in generation of Tol-DC that suppressed T cell responses and selectively promoted Treg generation. The combination of MSC synergized the tolerogenic capacity of Tol-DC in inhibition of T cell responses. In murine collagen-induced arthritis (CIA) model, we demonstrated that progression of arthritis was inhibited with administration of RelB gene-silenced Tol-DC or MSC. This therapeutic effect was remarkably enhanced with concurrent treatment of combination Tol-DC and MSC as demonstrated by improved clinical symptoms, decreased clinical scores and attenuated joint damage. These therapeutic effects were associated with suppression of CII-specific T cell responses, polarization of Th and inhibition of proinflammatory cytokines, and reduced cartilage degeneration. This study for the first time demonstrates a new approach to treat autoimmune inflammatory joint disease with concurrent treatment of RelB gene-silenced Tol-DC and MSC.

Rheumatoid arthritis (RA) is an autoimmune disease characterized by progressive immune-mediated joint deterioration. Chronic inflammation of the synovium and the presence of RA synovial fibroblasts (RASFs) undergo hyperplasia and invade cartilage and bone. RASFs produce proinflammatory cytokines, such as IL-1 and TNF-α, which provide further stimulation for ongoing inflammation^1^. Furthermore, enzymes including stromelysin and collagenase are produced, which contribute to deterioration of cartilage and bone. Current treatments are not antigen specific, and they are associated with various adverse effects as well as significant health-care costs.

We^2^,^3^ and others^4^,^5^ have demonstrated that Tol-DC^6^, professional antigen-presenting cells, are potent antigen-specific immune regulators that hold great promise in immunotherapy of autoimmune diseases. RelB gene can guide differentiation of naïve T cells into a Th1 cytotoxic/inflammatory state. We have previously demonstrated in a collagen-induced arthritis (CIA) animal model that DC did not mature when treated with a synthetic RelB inhibitor, and this prevented disease progression.

In contrast to Tol-DC, mesenchymal stem cells (MSC) are defined as pluripotential cells capable of self-renewal and differentiation into adipose, osteoblast as well as chondrocytic lineages^7^. More importantly, in conditions of joint injury, MSC are capable of selectively homing to damaged joints and undergoing chondrocytic differentiation as well as stimulating repair processes^8^,^9^. It is known that inflammatory conditions are associated with resistance to tolerance induction. Therefore, we hypothesize that suppressing inflammation by inhibiting cartilage degeneration with MSC while concurrently inducing antigen-specific immune modulation with Tol-DC will result in synergistic suppression of RA in a murine CIA model.

In this study, we take advantage of two technical advances: tailor-made Tol-DC using siRNA and *in vitro-*manipulated MSC. We are able to demonstrate the development of a novel approach to combine Tol-DC and MSC as immunotherapy for arthritis. We further delineate the synergistic function of Tol-DC and MSC in immune modulation as well as the suppressed cartilage degeneration after injury occurred. This is the first demonstration of RA treatment using a combination approach of inhibiting cartilage degeneration and promoting immunomodulation by concurrently treating CIA with Tol-DC and MSC.

## Results

### Tol-DC generated with siRelB

**Figure 1:**
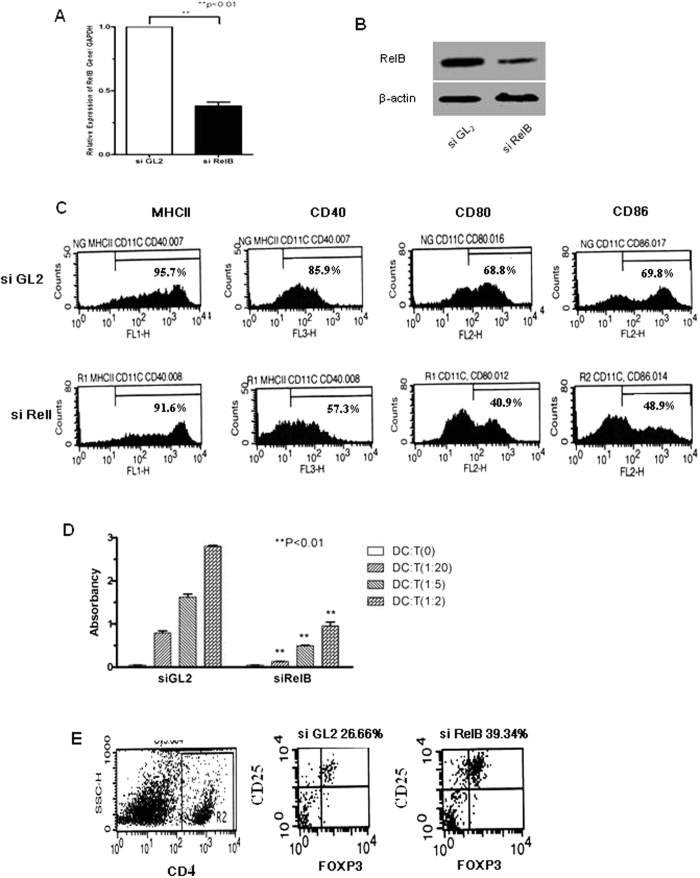
Alteration of phenotype and function of DC after gene silencing of RelB. (**A**,**B**) Gene silencing efficacy. DC were cultured from bone marrow of DBA mice as described in Materials and Methods. 7-day-cultured DC were transfected with RelB siRNA and control siRNA (siGL2). 48 h after transfection, DC were harvested and the expression of RelB was detected by (**A**) qRT-PCR and (**B**) Western blot. Cropped gel images are shown and full-length gel images are included in Supplementary Figure S1. (**C**) Maturation of DC after gene silencing of RelB. DC were generated and transfected with RelB and control siRNA as described in (**A**). DC were stained with antibodies against MHC II, CD40, CD80 and CD86, respectively. The expression of above molecules was detected by flow cytometry. (**D**) APC function of DC after gene silencing of RelB. After gene silencing, DC from DBA were used as stimulator cells and incubated with allogeneic T cells from BALB/c mice for 3 days in an MLR assay. T cell proliferation was detected by MTT assay. (**E**) Generating Treg cells by RelB-silenced DC. The RelB and control siRNA-transfected DC were incubated with allogeneic T cells, as described in (**D**), for 7 days. The T cells were isolated and stained with antibodies CD4, CD25 and Foxp3, respectively. After gating CD4 positive cells, the expression of CD25 and Foxp3 was detected by flow cytometry.

RelB is a critical factor controlling DC maturation and function^7^,^8^. To test if RelB gene silencing can alter DC, we cultured DC from bone marrow of DBA mice following gene silencing using siRNA. The expression of RelB in DC was significantly repressed after gene silencing as detected by qRT-PCR (Fig. 1A) and Western blot (Fig. 1B). Next, we examined DC maturation after gene silencing of RelB. The maturation markers MHC II, CD40, CD80 and CD86 in DC silenced with siRelB were markedly decreased, as compared with control siRNA siGL2 transfected DC (Fig. 1C). To determine the APC function of DC after gene silencing, an MLR assay was conducted using gene silenced DC from DBA as stimulators. T cell proliferation stimulated by RelB-silenced DC was significantly suppressed in a dose-dependent manner (Fig. 1D). It was observed that RelB-silenced DC promoted generation of CD4 + CD25 + Foxp3+ Treg cells *in vitro* (Fig. 1E). These data suggest that silencing RelB in DC can enhance tolerogenic properties.

### Generation and characterization of MSC

**Figure 2:**
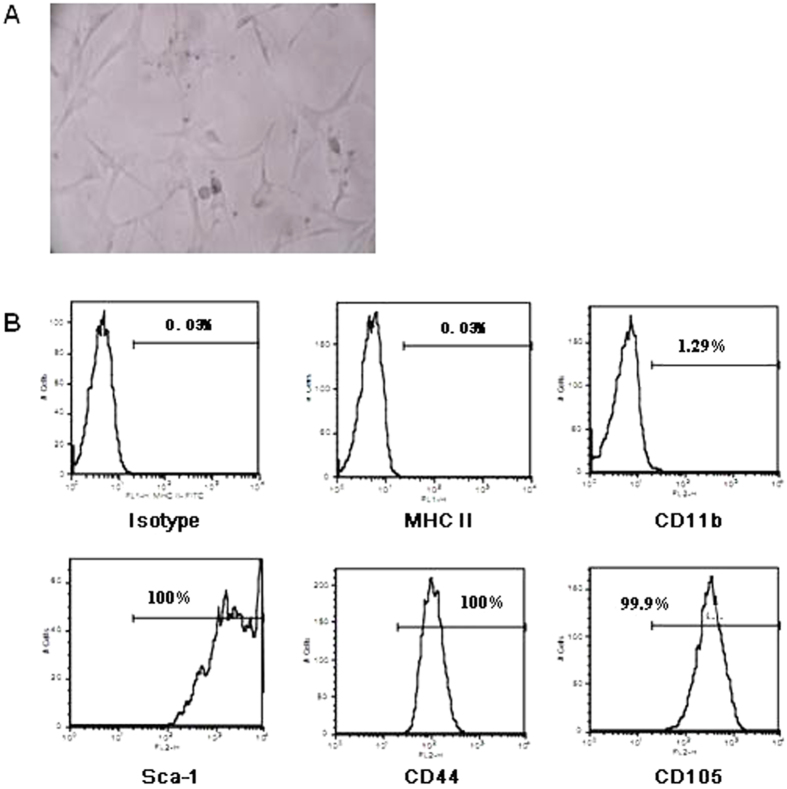
Preparation and identification of MSC. MSC were cultured from bone marrow of DBA mice as described in Materials and Methods. (**A**) Morphology of cultured MSC. After 21-day culture, MSC were observed under microscope. (**B**) Phenotypes of MSC. 21-day-cultured MSC were harvested and stained with MHC II, CD11b, Sca-1, CD44 and CD105, respectively. The expression of above molecules was detected by flow cytometry.

MSC were cultured from bone marrow of DBA mice as described in Materials and Methods. Cultured MSC showed typical morphology as displayed in Fig. 2A. They showed high expression of Sca-1, CD44 and CD105, but were negative in MHC II and CD11b (Fig. 2B), suggesting MSC were successfully generated from DBA mice.

### Synergistic effects of Tol-DC and MSC in treatment of CIA

**Figure 3:**
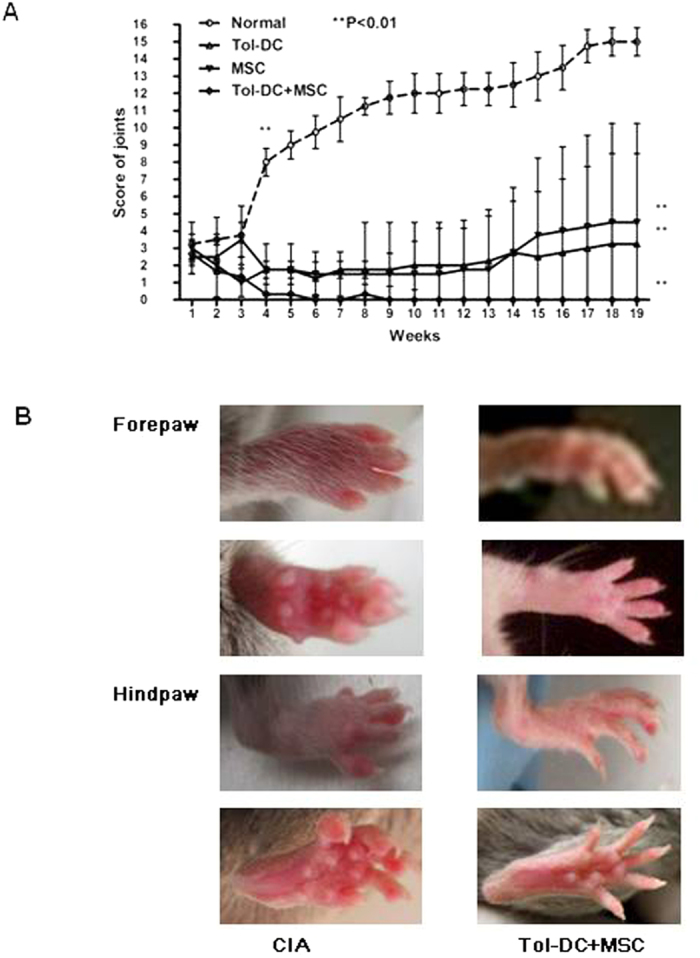
Synergistic effects of Tol-DC and MSC in treatment of CIA. (**A**) Score of joints. CIA was established in DBA mice as described in Materials and Methods. CIA mice were treated with Tol-DC, or MSC, or combination. The scores of diseased joints were measured and observed for 19 weeks. (**B**) Symptoms of CIA mice. Symptoms in fore paws and hind paws at the end point of observation are displayed.

Increasing evidence demonstrates that Tol-DC can induce immune tolerance while MSC possesses regenerative properties^9^, suggesting a synergistic therapeutic effect in treating CIA characterized by autoimmunity and joint damage. To test this hypothesis, we treated CIA mice with Tol-DC, or with MSC, or with a combination of both. The scores of diseased joints were significantly decreased in the CIA mice that received treatment with Tol-DC or MSC; whereas combination treatment with both cells prevented the development of CIA (Fig. 3A). Moreover, while CIA mice developed severe symptoms of arthritis, treatment with combined Tol-DC and MSC remarkably attenuated symptoms of CIA (Fig. 3B).

### Immunomodulation by Tol-DC and MSC

**Figure 4:**
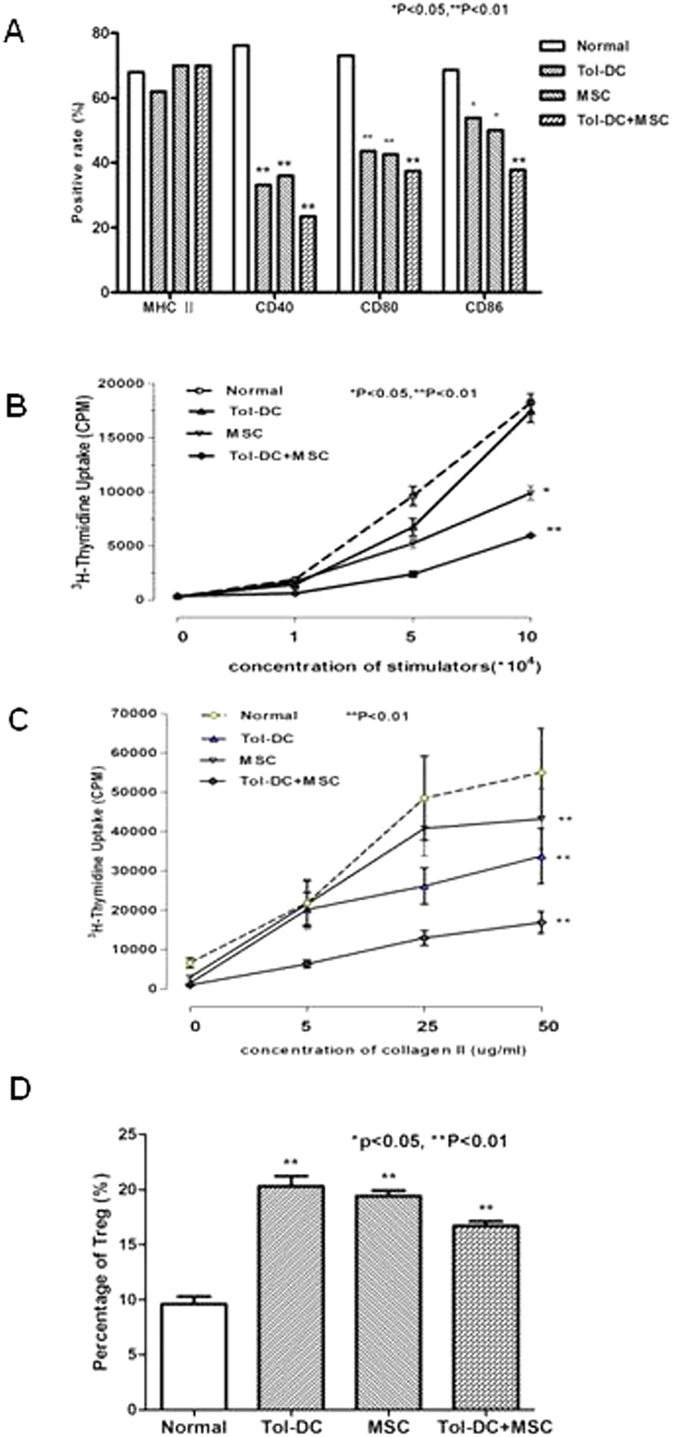
Synergistic effects of Tol-DC and MSC in immunomodulation. (**A**) Maturation of splenic DC in CIA mice. CIA mice were developed and treated with Tol-DC, or MSC, or combination, as described above. The splenic DC were isolated and stained with antibodies against MHC II, CD40, CD80 and CD86, respectively. The expression of above molecules was detected by flow cytometry. (**B**) DC function in DBA mice. Splenic DC (isolated from CIA mice) treated with Tol-DC, or MSC, or a combination were used as stimulator cells and incubated with allogeneic T cells for 3 days in an MLR assay. T cell proliferation was detected by 3-H incorporation assay. (**C**) Antigen-specific response. T cells were isolated from CIA mice treated with Tol-DC, or MSC, or combination. T cell proliferation was tested in the presence of collagen II at indicated concentrations. After 3 days of incubation, T cell proliferation was detected by 3-H incorporation assay. (**D**) Treg cells in CIA mice. T cells were isolated from CIA mice treated with Tol-DC, or MSC, or combination. T cells were stained with antibodies CD4, CD25 and Foxp3, respectively. After gating CD4 positive cells, the expression of CD25 and Foxp3 was detected by flow cytometry.

It has been reported that both Tol-DC^6^ and MSC^10^ suppress immune responses. To test if a combination of the two cell types can synergistically achieve immune modulation, we examined splenic DC in CIA mice after treatment with Tol-DC, or with MSC, or with combination treatment. The splenic DC displayed less maturity after treatment with Tol-DC or MSC alone, but combination treatment further prevented DC maturation as evidenced by the repression of DC maturation markers CD40, CD80 and CD86 (Fig. 4A). The splenic DC, isolated from CIA mice treated with Tol-DC or MSC showed impaired antigen-presenting function, whereas combination treatment further inhibited the APC ability of DC (Fig. 4B). Next, we assessed the antigen-specific response of T cells in the presence of collagen II. T cells, isolated from CIA mice treated with Tol-DC, MSC, or combination, showed suppressed proliferation (Fig. 4C). Finally, we examined Treg cells in CIA mice. The non-treated mice showed a similar level of Treg (9.6%), yet treatment with Tol-DC or MSC significantly increased the Treg numbers (20.3% and 19.4%, respectively). Combination treatment with Tol-DC and MSC further upregulated the generation of Treg cell up to 16.7% (Fig. 4D). Taken together, these data imply that Tol-DC and MSC can work synergistically to alter immunomodulation and suppression of immune responses in CIA mice.

### Polarization of Th and inhibition of proinflammatory cytokines by Tol-DC and MSC

**Figure 5:**
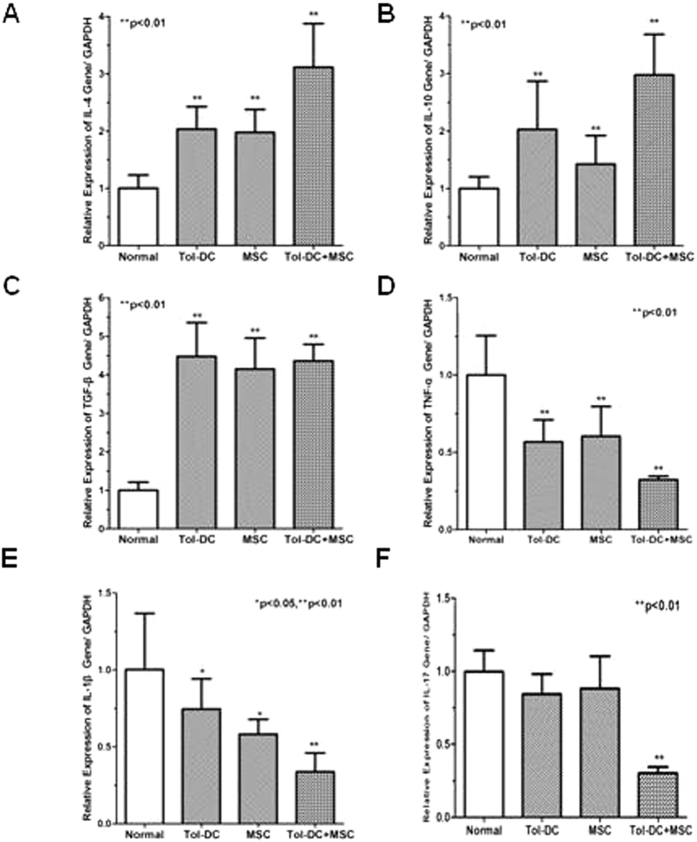
Alteration of proinflammatory cytokines in CIA mice after combination treatment with Tol-DC and MSC. CIA mice were treated with Tol-DC, or MSC, or combination. T cells were isolated and the mRNA was prepared. Expression of IL-4 (**A**), IL-10 (**B**), TGF-β (**C**), TNF-α (**D**), IL-1β (**E**) and IL-17 (**F**) were detected by qRT-PCR.

There is some evidence that Th switch and upregulation of IL-17 and proinflammatory cytokines play important roles in the onset and progress of autoimmune arthritis^11^. To assess if Tol-DC and MSC can influence cytokines in CIA mice, we tested cytokine production of T cells isolated from CIA mice after treatment with Tol-DC, or MSC, or a combination. As shown in Fig. 5, expressions of IL-4, IL-10 and TGF-β were significantly increased in the mice treated with Tol-DC and MSC, as compared with T cells from non-treated CIA mice (Fig. 5A,B,C). In contrast, CIA mice displayed high levels of TNF-α and IL-1β, which were significantly suppressed by the treatment of Tol-DC and MSC (Fig. 5D,E). In addition, the expression of IL-17, upregulated in the CIA mice, was also remarkably suppressed by Tol-DC and MSC combination treatment (Fig. 5F). These data highlight that Tol-DC and MSC upregulate Th2 and Th3 while suppressing IL-17 and proinflammatory cytokines in CIA mice.

### Attenuating joint damage and suppression of cartilage degeneration by Tol-DC and MSC

**Figure 6:**
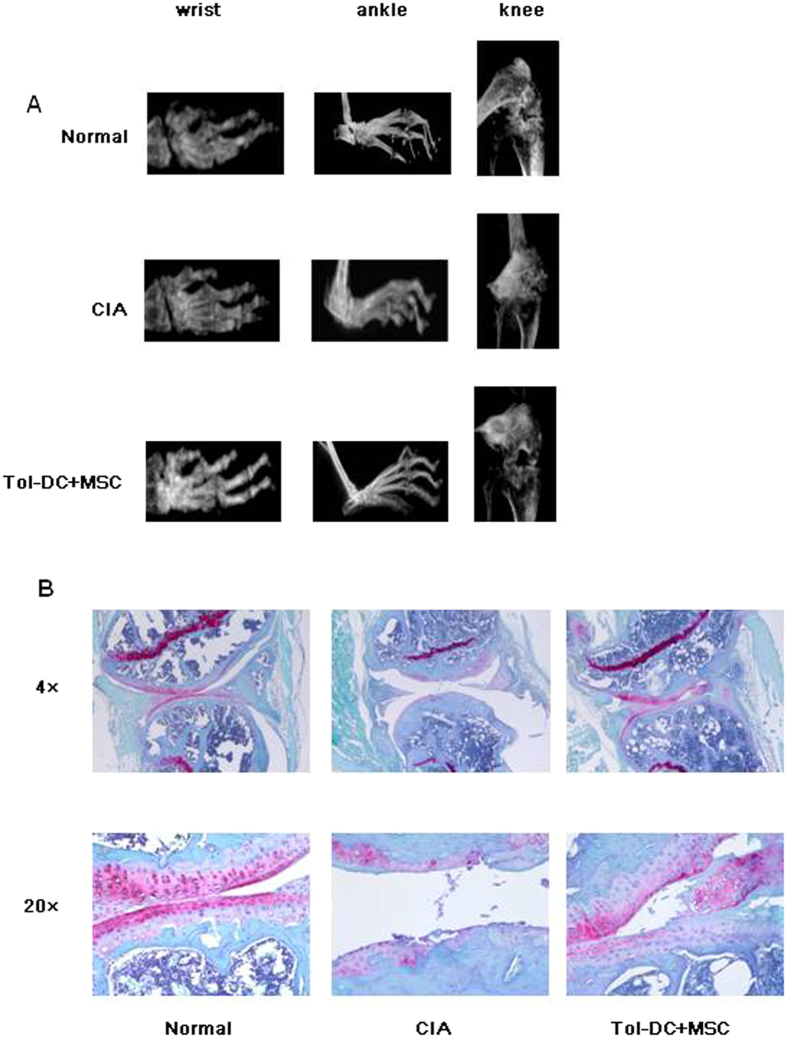
Preventing joint damage and cartilage degeneration by concurrent treatment of Tol-DC and MSC. (**A**) Joint damage in CIA mice. CIA was established in DBA mice as described in Materials and Methods. CIA mice were treated with Tol-DC and MSC. The joints in wrists, ankles and knees were scanned by microCT. (**B**) Cartilage degeneration in CIA mice. CIA mice were treated with Tol-DC and MSC. Knee joints from CIA were collected, fixed and sectioned. Sections were stained with Safranin O and Fast Green and observed under microscope.

Joint damage in RA results in adverse effects on bone remodeling, skewing the balance toward cartilage degeneration^12^. The therapeutic effect of Tol-DC and MSC on joint damage was determined by μCT of bone structures, following evaluation of cartilage degeneration. Significant decrease of damage in all joints, including wrists, ankles, and knees, was observed in CIA mice, whereas treatment with Tol-DC and MSC showed a protective effect on inflammation-induced bone loss (Fig. 6A). Significant cartilage loss occurred in knees of CIA mice; in contrast, treatment with Tol-DC and MSC attenuated cartilage degeneration (Fig. 6B). These data confirm a strong synergistic protective effect of Tol-DC and MSC on both joint damage and cartilage degeneration in CIA mice.

## Discussion

Rheumatoid arthritis (RA) is a chronic autoimmune condition characterized by non-specific, usually symmetric inflammation of the peripheral joints, resulting in progressive destruction of articular and periarticular structures. One of the hallmark pathologies of RA is thickening and swelling of synovial tissue, primarily as a result of T cell production of inflammatory factors^9^. Up to 50% of the infiltrating leukocytes in the synovium are T-lymphocytes, primarily CD4+ T cells with an activated/memory phenotype^10^,^11^, expressing a Th1 bias^11^,^12^. The most established experimental system used to study RA is the collagen-induced arthritis (CIA) model, in which DBA mice are immunized with heterogonous collagen type II (CII) proteins in the presence of an adjuvant to stimulate anti-synovial autoimmunity. The CIA model resembles the clinical progression of RA from a histological perspective and is immunologically similar since it is MHC II-linked and involves both autoreactive T cell and autoantibody responses^13^. Similar to RA, CIA is associated with a Th1/Th17 immune profile (i.e. IFN-γ, IL-2, IL-12)^14^ and its progression is inhibited by CD4^+^ CD25^+^ Treg cells^15^. The CIA model has been extensively used in the development of novel drug therapy for RA. Thus, we used this model to test a new approach of combination immunotherapy using Tol-DC and MSC.

Clinical treatment of RA involves early initiation of Disease Modifying Anti-Rheumatic Drug (DMARD) therapy following diagnosis with subsequent optimization of drug therapy in order to have a greater beneficial impact on disease outcome^15^. DMARD therapy is antigen nonspecific and includes known immune suppressants such as methotrexate, leflunomide, hydroxychloroquine, sulfasalazine, and corticosteroids. The introduction of “biological DMARD”, such as Enbrel and Remicade, led to a major improvement in quality of life for RA patients; however, these drugs are limited by cost, non-cure of the disease, and adverse effects such as heightened risk of infection^16^. Despite promising animal data, to date antigen-specific treatments of RA have not been clinically successful. While approaches such as intravenous immunoglobulin^16^, oral tolerance^17^, and tolerogenic peptide therapy^18^ have demonstrated promising results in various models, clinical trials have yielded results that are mediocre at best. Therefore, more robust immunotherapy is of great clinical significance.

We have manipulated DC possessing immune regulatory functions, which are collectively termed “tolerogenic DC” (Tol-DC)^19^,^20^. We have demonstrated that Tol-DC possess the following tolerogenic properties: (1) immature phenotypes^18^, (2) decreased allostimulatory capacity^21^,^22^, (3) impaired costimulatory function^21^,^22^, (4) ability to polarize Th2 differentiation^19^, and (5) capability to generate Treg^21^,^23^ and to transfer tolerance to naïve mice^24^. Generation of Tol-DC *in vitro* can be performed by culturing DC in conditions that inhibit maturation of these cells. We have also generated “tailor-made” Tol-DC by gene transfecting FasL^21^,^25^, or by manipulating DC with immunosuppressants^19^. Using the technique of RNA interference (RNAi), a process by which double-stranded RNA selectively inactivates homologous mRNA transcripts, we have been able to successfully generate Tol-DC by knocking down selected immune stimulatory genes. Our initial silencing of IL-12 in DC demonstrated that immune modulation can be achieved through RNAi^7^. We subsequently reported that silencing RelB in DC led to expansion of Treg cells and tolerance induction^26^. In other experiments, we demonstrated that *ex vivo* silencing of CD40 in DC can ameliorate RA^22^ and allergic diseases^27^. Recently, we developed a new method specifically targeting DC using immunoliposome^28^, making RNAi-based immunotherapy more clinically applicable. In this study, we demonstrated that gene silencing of RelB in DC resulted in multiple tolerogenic properties including suppression of the immune response both *in vitro* and *in vivo*.

In a previously study, we successfully developed a new antigen-specific immunotherapy for autoimmune arthritis^4^,^29^,^30^ using Tol-DC. We further demonstrated, in a murine model of RA, that *in vitro* or *in vivo* gene-silenced Tol-DC possess therapeutic effects in treating autoimmune arthritis by inducing antigen-specific immune suppression and generating Treg cells^2^. Such approaches show great promise in the treatment of RA during early stages, but have less clinical impact once cartilage damage has reached a significant level. Therefore, in this study, our intent was to develop a strategy that concurrently blocks the underlying cause of joint damage and promotes healing. We aimed to suppress the autoimmune process using Tol-DC and to inhibit cartilage degeneration by administrating MSC.

MSC are stromal-like cells, originally discovered in the bone marrow as non-hematopoietic cells capable of self-renewal and, upon induction, differentiation into chondrocyte, osteoblast, and adipocyte lineages. Although MSC may be derived from a variety of sources, the majority of efforts have focused on bone marrow-derived MSC. MSC are defined as plastic-adherent cells possessing a fibroblastoid-like morphology that are negative for hematopoietic markers (CD34, CD45), positive for CD73, CD90 and CD105 (Fig. 2), and have the ability to differentiate into bone, cartilage, and adipose tissue^31^. As a biological treatment for autoimmunity, MSC appear to be an ideal cellular population owing to the following properties: they express CXCR4 and several other chemokine receptors, allowing their migration to areas of tissue injury, thereby permitting intravenous administration, and they secrete growth factors such as IGF-1, HGF-1, bFGF, allowing MSC to induce an indirect healing function^23^. It has been reported that MSC have immune privilege characteristics that permit the use of allogeneic MSC, thereby avoiding the need for HLA-matching^24^, while the immune regulatory activities of MSC may suppress ongoing autoimmunity^32^. In our previous study, we discovered a synergistic immunoregulatory feedback loop between Tol-DC and Treg, in which Tol-DC promotes Treg generation while Treg facilitates DC retaining Tol-DC properties^18^. It would be interesting whether MSC may enhance the Tol-DC/Treg immunoregulatory feedback loop. Moreover, the ability of MSC to directly differentiate into damaged tissues suggests the possibility of allowing tissue regeneration after initial damage has occurred^26^.

The above characteristics of MSC have prompted numerous preclinical and clinical investigations. RA patients have MSC with shorter telomeres and limited replicative ability in the synovial fluid. Some investigators to postulate that MSC are continually made locally in an attempt to compensate for ongoing injury. Supporting this possibility are data from *in vitro* experiments using RA-derived MSC showing these cells alone, or after differentiation into chondrocytes, are able to inhibit CII-specific T cell responses. In the context of CIA, treatment with MSC has induced remission with regeneration of damaged joint tissue. Other investigators have demonstrated that systemic administration of MSC not only inhibits CIA pathology but also induces CII-specific Treg cells. While the *in vivo* effect of MSC in the context of RA is still unclear, numerous animal studies have supported its beneficial role in a variety of immune-mediated pathologies such as EAE, transplant rejection, diabetes^29^, a mouse model of SLE^27^, and autoimmune enteropathy^28^.

In the current study, we take advantage of two technical advances: tailor-made Tol-DC using RelB-siRNA and *in vitro*-manipulated MSC. We have been able to demonstrate that concurrent treatment of RelB-silenced Tol-DC (that provide antigen-specific immune suppression) *and* MSC (that inhibit degeneration of joints) can be used to effectively suppress autoimmune arthritis. This finding may be used to develop new MSC-based therapies for autoimmune arthritis.

## Materials and Methods

### Animals

Male DBA/1 LacJ and BALB/c mice, 5 weeks of age, were purchased from Jackson Laboratories (Bar Harbor, ME) and kept in filter-top cages in the Animal Care and Veterinary Services Facility at the University of Western Ontario. All experimental protocols were in accordance with the approved guidelines for safety requirements of the Canadian Council for Animal Care Guidelines, and were approved by the Animal Use Committee, University of Western Ontario. Mice were provided food and water and allowed to settle for 2 weeks before initiation of experiments, which had ethical approval from the university’s review board. Male DBA/1 LacJ mice were divided into 4 groups (CIA, CIA pretreated with Tol-DC, CIA pretreated with MSC, and CIA pretreated with combined Tol-DC and MSC) with each group having 5 mice.

### CIA Model

DBA/1 LacJ mice, 7 weeks of age, were intradermally immunized (Day 0) at several sites in the base of the tail with 100 μg (100 μl) chicken type II collagen (CII) (Sigma-Aldrich, St. Louis, MO) and mixed with an equal volume of complete Freund’s adjuvant (CFA) (Sigma-Aldrich). On day 21 after priming, the mice received an intraperitoneal booster injection with 100 μg (100 μl) CII mixed with an equal volume of incomplete Freund’s adjuvant (IFA) (Sigma-Aldrich). Mice were examined visually three times per week for the appearance of arthritis in the peripheral joints, and arthritis score index for disease severity was given as follows: 0 – no evidence of erythema and swelling; 1 – erythema and mild swelling confined to the mid-foot (tarsals) or ankle joint; 2 – erythema and mild swelling extending from the ankle to the mid-foot; 3 – erythema and moderate swelling extending from the ankle to the metatarsal joints; 4 – erythema and severe swelling encompass the ankle, foot, and digits. Scoring was done by two independent observers without knowledge of the experimental and control groups.

### DC cultures

At Day 0, bone marrow cells were flushed from the femurs and tibias of DBA/1 LacJ mice, washed and cultured in 6-well plates (Corning Life Sciences, Corning, NY) at 4 × 10^6^ cells/well in 4 ml of complete medium (RPMI 1640 supplemented with 2 mM L-glutamine, 100 U/ml penicillin, 100 μg of streptomycin, 50 μM 2-ME, and 10% FCS (all from Life Technologies, Burlington, ON, Canada) supplemented with recombinant GM-CSF (10 ng/ml; PeproTech, Rocky Hill, NJ) and recombinant mouse IL-4 (10 ng/ml; PeproTech). All cultures were incubated at 37 °C in 5% humidified CO_2_. Non-adherent cells were removed after 48 h of culture (Day 2) and fresh medium was added every 48 h.

### RelB gene-silencing DC

siRNA sequences were selected according to the method of Elbashir *et al*.^30^. The siRNA sequence specific for RelB (GGAAUCGAGAGCAAACGA) was selected and transfected to DC. DC were generated from bone marrow progenitor cells as previously described^6^,^7^. 24 h before transfection (day 4), DC were plated to be 60–90% confluent on the day of transfection. On day 5, 2 μg of RelB siRNA and 1 μl of lipofectamine 2000 (Invitrogen, Carlsbad, CA) were separately diluted with serum-low medium Opti-MEM using ½ of the transfection volume (125 μl). The diluted RelB siRNA was added to the diluted lipofectamine 2000 after incubation at room temperature for 5 min, mixed rapidly and incubated in total volume of 250 μl of the medium at room temperature for 25 min. The culture medium from the DC was aspirated, and the RelB siRNA-lipofectamine 2000 mixture was added carefully to the DC. Mock controls were transfected with GL2 siRNA-lipofectamine 2000 mixture. After 4-h incubation, an equal volume of RPMI 1640 (250 μl) supplemented with 20% FCS was added to the cells. 48 h after the start of transfection (day 7), DC were washed and harvested. 7 days and 14 days after priming with CII, different groups of mice with 5 animals per group were IV injected with RelB siRNA-transfected DC or GL2 siRNA-transfected DC at a dose of 2 × 10^6^ cells per mouse.

### Maturation of DC

DC were generated from bone marrow progenitor cells as previously described. 48 h after transfection with siRelB or siGL2, DC were harvested and stained with MHC II-FITC, CD11C-Per CP, CD40-PE and CD86 PE. Flow cytometry analysis was performed in a FACScan II (Becton Dickinson, San Jose, CA) system using FACSDiva software (Becton Dickinson).

### MSC culture

At Day 0, bone marrow cells were flushed from the femurs and tibias of DBA/1 LacJ mice, washed and cultured in 75 cm flask (Corning Life Sciences) at 2 × 10^7^ cells/flask in 15 ml of complete medium (DMEM supplemented with 2 mM L-glutamine, 100 U/ml penicillin, 100 μg of streptomycin, 50 μM 2-ME, and 10% FCS (all from Life Technologies). All cultures were incubated at 37 °C in 5% humidified CO_2_. Non-adherent cells were removed after 72 h of culture (Day 3) and fresh medium was added every 72 h. Bone marrow original cells were passaged and harvested whenever they achieved 70–80% confluence, and harvested about 21 days later. Monocytes were depleted by negative selection for CD11b using magnetic-activated cell sorting (eBioscience, San Diego, CA). After detecting the purity of MSC by markers positive for Sca-1, CD44 and CD105, negative for MHC II and CD11b, different groups of mice with 5 animals per group were IV injected with MSC at a dose of 2 × 10^6^ cells per mouse once a week for 4 weeks after receiving a booster of CII.

### qRT-PCR

Total RNA was extracted from cells using Trizol (Invitrogen). 3 μg total RNA was used to synthesize the cDNA with oligdT and reverse transcriptase (Invitrogen) in 20 ul reaction volume. Primers used for the amplification of murine Rel-B, IL-4, IL-10, TGF-β, TNF-α, IL-1β, IL-17 and GAPDH were as follows^8^: Rel-B, 5′-GGT GAC GGC GTG CCT GGT GTG-3′ (forward) and 5′-ACG GCC CGC TCT CCT TGT TGA TTC-3′ (reverse); IL-4, 5′-AGC TAG TTG TCA TCC TGC TCT TC-3′ (forward) and 5′-AGC ATG GTG GCT CAG TAC TAC G-3′ (reverse); IL-10, 5′-TGC TAT GCT GCC TGC TCT TAC TGA C-3′ (forward) and 5′-AAT CAC TCT TCA CCT GCT CCA CTG-3′ (reverse); TGF-β, 5′-AAC AAA CTC CAC GTG GAA ATC AAC-3′ (forward) and 5′-CTT GCG ACC CAC GTA GTA GAC GAT-3′ (reverse); TNF-α, 5′-GCC TCT TCT CAT TCC TGC TTG TGG-3′ (forward) and 5′-CCC GTT ATC TCC CCT TCA TCT TCC-3′ (reverse); IL-1β, 5′-ACC TGG GCT GTC CTG ATG AGA G-3′ (forward) and 5′-CCA CGG GAA AGA CAC AGG TAG C-3′ (reverse); IL-17, 5′-CCT GGG TGA GCC GAC AGA AGC-3′ (forward) and 5′-CCA CTC CTG GAA CCT AAG CAC-3′ (reverse); GAPDH, 5′-TGA TGA CAT CAA GAA GGT GGT GAA-3′ (forward) and 5′-TCC TTG GAG GCC ATG TAG GCC AT-3′ (reverse). Quantative real-time PCR was performed in 20 μl of reaction volume containing 0.2 μmol/L primers, 1U Taq DNA polymerase under the following conditions: 95 °C for 30 sec, and then 95 °C for 5 sec, 58 °C for 45 sec for 40 cycles, and then 95 °C for 15 sec, 60 °C for 1 min, 95 °C for 15 sec.

### Western blot

Cells were harvested, washed twice with ice-cold PBS, re-suspended in protein lysis buffer with complete protein inhibitor, and then the container was kept on ice for 30 min. Lysed cells were centrifuged at 15000 × RPM for 20 min at 4 °C and the supernatant was collected and stored at −80 °C for future use. Protein concentration was determined by Bio-Rad protein assay and 50 μg lysate was separated on 12% SDS-PAGE, transferred to nitrocellulose membrane, blocked with 5% fat-free milk and 3% BSA in TBS-T (0.25% Tween 20), probed with a mouse anti-mouse RelB mAb (Santa Cruz Biotechnology, Paso Robles, CA) and anti-β-Actin Ab (Sigma, St. Louis, MO) according to the manufacturer’s instructions, and visualized by an ECL assay (Pierce, Rockford, lL).

### Mixed Leukocyte Reaction (MLR)

Spleenic DC were positively selected using CD11c magnetic-activated cell sorting (eBioscience). Sorted DC were triplicate seeded in 96-well plate (Corning) for use as stimulator cells. Splenic T cells from BALB/c mice were isolated by gradient centrifugation over Ficoll-Paque (Amersham Pharmacia Biotech, Quebec, Canada) and added as responders (5 × 10^5^ cells/well). The mixed lymphocytes were cultured at 37 °C for 72 h in 200 μl of RPMI 1640 supplemented with 10% FCS, 100 U/ml of penicillin, and 100 μg/ml of streptomycin. The co-cultures were pulsed with 1 μCi/well of ^3^H-thymidine (Amersham) for the last 16 h of culture. The cells were harvested onto glass fiber filters, and the radioactivity incorporated was detected using a Wallac Betaplate liquid scintillation counter (Beckman, Fullerton, CA).

### CII-specific T cell proliferation

T cell proliferative responses to CII in subsequent groups of mice were measured with a standard microtiter assay. Following CII immunization, proliferative responses could be detected for several weeks. Immune cells from splenic T cells collected from 4 different groups were seeded to a 96-well flat-bottom microtiter plate (Corning) in triplicates at 5 × 10^5^/well and mixed with serial dilutions of CII with concentrations ranging from 5 to 50 μg/well. Following 72 h incubation, 1 μCi of [^3^H] thymidine (Amersham) was added to each well for 16 h. Using an automated cell harvester, the cells were collected onto glass microfiber filter, and the radioactive labeling incorporation was measured by a Wallac Betaplate liquid scintillation counter.

### Histology

The paws from experimental and control mice were removed and joint tissues were fixed in 10% (wt/vol) neutral buffered formalin in 0.15 M PBS (pH 7.4). After decalcification by Decalcifier I solution (Leica Biosystems, Winnipeg, MB, Canada) overnight and subsequent dehydration in a gradient of alcohols, tissues were processed for paraffin embedding in paraplast (BDH, Dorset, UK) as routine procedure. Serial paraffin sections throughout the joint were cut at 5-μm thickness on a microtome, heated at 60 °C for 30 min, and deparaffinized. Hydration was done by transferring the sections through the following solutions: triple to xylene for 6 min, and then for 2 min to 100% ethanol twice, 95% ethanol, and 70% ethanol. Sections were stained with Safranin O and Fast Green and mounted on glass slides.

### Treg cell determination

19 weeks after priming with CII, different groups of mice with 5 animals per group were sacrificed, Immune cells from splenic T cells collected from 4 different groups were stained with CD4-Per CP, CD25-PE and FOXP3-FITC, Flow cytometry analysis was performed in a FACScan II (Becton Dickinson, San Jose, CA- BD) system using FACSDiva software (BD).

### Microcomputed tomography

Microcomputed tomography (microCT or μCT) of excised joints including wrists, knees and ankles was carried out by a Skyscan 1076 CT scanner (Skyscan, Aartselaar, Belgium), following the general guidelines used for assessment of bone microarchitecture in rodents using microCT. Briefly, scanning was done at 50 kV, 200 uA using a 0.5-mm aluminum filter at a resolution of 9 um/pixel. CT Analyzer software was MicroView.

### Statistical analysis

Data are expressed as mean ± SEM. Differences between different groups of mice were compared using the Mann-Whitney U test for nonparametric data. A *P* value less than 0.05 was considered significant.

## Additional Information

**How to cite this article**: Li, R. *et al*. Synergistic suppression of autoimmune arthritis through concurrent treatment of tolerogenic DC and MSC. *Sci. Rep.* **7**, 43188; doi: 10.1038/srep43188 (2017).

**Publisher's note:** Springer Nature remains neutral with regard to jurisdictional claims in published maps and institutional affiliations.

## Supplementary Material

Supplementary Figure S1
