# Supplementary material for: Synergistic suppression of autoimmune arthritis through concurrent treatment with tolerogenic DC and MSC
Source: Sci Rep. 2017 Feb 23;7:43188. doi: 10.1038/srep43188 (PMC5322386; doi:10.1038/srep43188)

Synergistic suppression of autoimmune arthritis through concurrent treatment with tolerogenic DC and MSC

Rong Li[1,2], Yujuan Zhang[1,2], Xiufen Zheng[3], Shanshan Peng[1,2], Keng Yuan[1,2], Xusheng Zhang[3] and Weiping Min [1,2,3*]

Supplementary Information: Whole length gel image for Figure 1B

Supplementary Figure S1

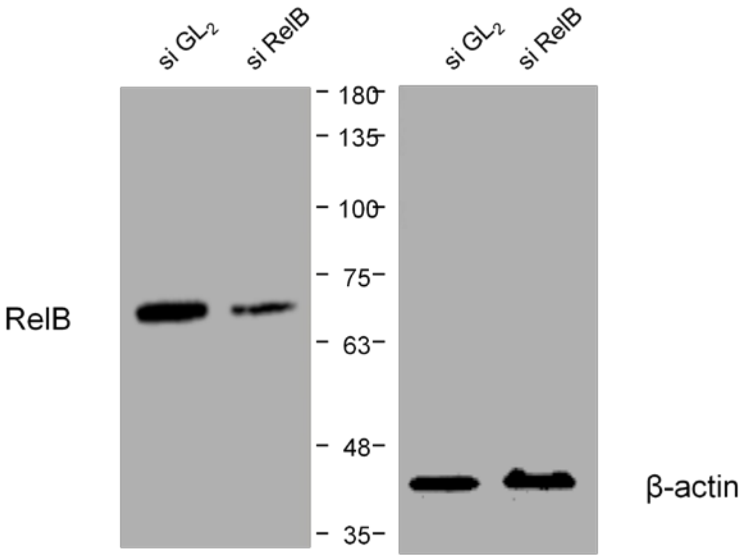

Supplement: Supplementary Figure S1 [file srep43188-s1.pdf]
